# Physical activity and sleep changes among children during the COVID-19 pandemic

**DOI:** 10.1038/s41746-024-01041-8

**Published:** 2024-03-16

**Authors:** Karnika Singh, Sarah C. Armstrong, Brooke E. Wagner, Julie Counts, Asheley Skinner, Melissa Kay, Jennifer S. Li, Svati Shah, Nancy Zucker, Cody Neshteruk, Mary Story, Lilianna Suarez, William E. Kraus, Alexandra R. Zizzi, Jessilyn Dunn

**Affiliations:** 1 Duke University Department of Biomedical Engineering, Durham, NC USA; 2 Duke Center for Childhood Obesity Research, Durham, NC USA; 3 Duke University Department of Pediatrics, Durham, NC USA; 4 Duke University Department of Population Health Sciences, Durham, NC USA; 5 Duke Global Health Institute, Durham, NC USA; 6 Duke University Molecular Physiology Institute, Durham, NC USA; 7 Duke University Department of Psychiatry and Behavioral Science, Durham, NC USA; 8 Duke University Department of Biostatistics, Durham, NC USA

**Keywords:** Risk factors, Prognostic markers

## Abstract

Daily routines, including in-person school and extracurricular activities, are important for maintaining healthy physical activity and sleep habits in children. The COVID-19 pandemic significantly disrupted daily routines as in-person school and activities closed to prevent spread of SARS-CoV-2. We aimed to examine and assess differences in objectively measured physical activity levels and sleep patterns from wearable sensors in children with obesity before, during, and after a period of school and extracurricular activity closures associated with the COVID-19 pandemic. We compared average step count and sleep patterns (using the Mann–Whitney *U* Test) before and during the pandemic-associated school closures by using data from activity tracker wristbands (Garmin VivoFit *3*). Data were collected from 94 children (aged 5–17) with obesity, who were enrolled in a randomized controlled trial testing a community-based lifestyle intervention for a duration of 12-months. During the period that in-person school and extracurricular activities were closed due to the COVID-19 pandemic, children with obesity experienced objectively-measured decreases in physical activity, and sleep duration. From March 15, 2020 to March 31, 2021, corresponding with local school closures, average daily step count decreased by 1655 steps. Sleep onset and wake time were delayed by about an hour and 45 min, respectively, while sleep duration decreased by over 12 min as compared with the pre-closure period. Step counts increased with the resumption of in-person activities. These findings provide objective evidence for parents, clinicians, and public health professionals on the importance of in-person daily activities and routines on health behaviors, particularly for children with pre-existing obesity. Trial Registration: Clinical trial registration: NCT03339440

## Introduction

Childhood obesity is a widespread health problem in the United States and is driven by a multitude of factors^1^. Physical activity (PA) and sleep are two especially important modifiable behaviors that support progression toward a healthy weight in children with pre-existing obesity^1,2^. Structured routines, such as school and extracurricular programs for children, may support consistent engagement in health behaviors. The structured days hypothesis posits that compared to unstructured days, structured school day activities provide opportunities for mandatory PA, reducing screen time, and enforcing waketime^3^. The daily structure and routine that are associated with structured school days extends beyond the mandatory in-school activities and can occur before and after school times, such as commuting to and from school, walking between school activities, having consistent bedtimes and waketimes, and having pre-set meal and snack times. Consistent with this hypothesis, several studies have also reported increased body mass index (BMI) over the summer months as compared with during the school year^4–7^, which further stresses the importance of structure and routine for child health behaviors and ultimately health and weight related outcomes.

The COVID-19 pandemic resulted in unexpected closures of schools and extracurricular programs. Recent literature indicates that for children globally, these changes resulted in decreased levels of PA, increased sedentary time, and disrupted sleep patterns^8–38^. Various studies in the pediatric population around the world used subjective data and/or short-term accelerometer data to demonstrate disruption of PA habits and sleep routines. Reported reductions in PA ranged from 10–90 min per day^29–32,34,36^, with studies reporting decline in both weekday and weekend PA^33^. Reported sleep behavior changes included delayed bedtimes (by 0.65 h), shifted sleep midpoints (by over 2 h), reduced sleep efficiency (by 2.09%) and prevalence of sleep disturbance in 54% children^28,34,36,39^.

These considerable differences in findings could be driven by differences in data collection methods and duration as well as time points in the pandemic during which data were collected. Importantly, during the time of the pandemic, the rate of increase of BMI in children doubled compared to the pre-pandemic period, with larger increases among children with obesity^40^. Children with obesity are already at a higher risk for engaging in less PA and shorter sleep durations, and would benefit from regular PA and consistent sleep patterns^41–44^, identifying a critical need for exploring the impact of the COVID-19 pandemic among this population. However, few studies have addressed PA and sleep routines as a result of the COVID-19 pandemic specifically among children with obesity, and have used subjective data to report changes in sleep and PA^11,28^.

Additionally, the majority of the PA and sleep data in children reported during the COVID-19 pandemic were collected using self-report measures, which have low levels of reliability when assessing PA and sleep patterns^45,46^, especially for children^47^. Studies using objective data to demonstrate these changes in children have been few with minimal days of data collection from activity trackers^31–37^. Some of the previous studies using accelerometer or activity tracker data included data collection ranging from 3 days to 6 weeks each from pre-pandemic and post-pandemic period. These included reports of a 43.3 min and a 2.09% per day decrease in PA and sleep efficiency respectively in Spanish preschoolers^34^, a 10 min decrease in moderate-to-vigorous PA (MVPA) and 124 min delay in bedtime in children in southeastern United States^36^, and a 15.32 min decline in daily MVPA in children from Italian primary schools^32^.

Objectively measured PA and sleep from wrist-worn wearable devices can provide more accurate and detailed information compared to subjective data, including step counts during specific times of the day and sleep timings. Such objective and longitudinally collected data have yet to be leveraged to explore the changes in health behaviors that may result from disrupted daily routines (e.g., school and community center closures) due to COVID-19. The findings may inform future decision-making about systematic changes that may affect the daily habits and routines of children with obesity.

Therefore, the purpose of this study was to examine objectively measured PA and sleep in children with obesity prior to, during, and after COVID-19-related closures of in-person school and extracurricular programs.

## Results

### Physical activity

**Fig. 1:**
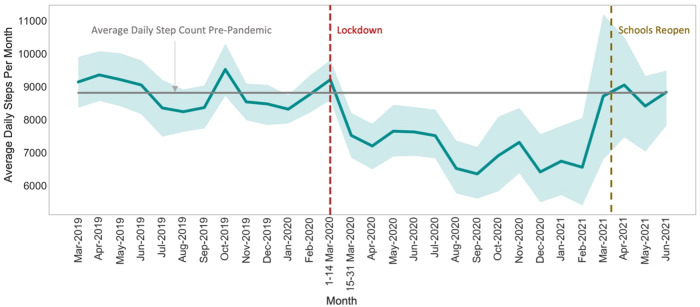
Average daily step count value with 95% confidence interval per month between March 2019 and June 2021. The horizontal gray line indicates the average daily step-count value between March 1, 2019 and March 14, 2020. The dashed vertical red and brown lines indicate the beginning of in-person public school closures and the return to in-person public schools, respectively, in North Carolina.

Of the 94 participants included in this analysis, 52 (55%) were female, 65 (69%) were in the age group 5–10 years (median age 9.7 ± 3.1), and 50 (53%) were assigned to the intervention group. The detailed demographics for study participants who were included in the analysis are shown in Table 1. Pre-closure daily step counts (averaged over the course of the month) ranged from 8239 to 9521 steps, with an average of 8810 ± 453 (*n* = 93) (Fig. 1). During-closure, the daily step counts dropped significantly from pre-closure to 7155 ± 669 steps per day (*p* < 0.05), with an average decline of 1655 steps (*n* = 53) (Fig. 1), and ranged from 6354 to 8711 steps. Post-closure, the daily step count averaged 8763 ± 325 steps (*n* = 8).

**Table 1:** Participant demographics

	Overall *n* (%)	Pre-closure *n* (%)	During-closure *n* (%)	Post-closure *n* (%)
Age Groups (years)				
5–10	65 (69%)	64 (69%)	37 (70%)	5 (63%)
11–13	16 (17%)	16 (17%)	9 (17%)	0 (0%)
14–18	13 (14%)	13 (14%)	7 (13%)	3 (37%)
Gender				
Male	42 (45%)	42 (45%)	25 (47%)	4 (50%)
Female	52 (55%)	51 (55%)	28 (53%)	4 (50%)
Race				
Other	37 (39%)	37 (40%)	21 (40%)	0 (0%)
Black/African American	32 (34%)	31 (33%)	21 (40%)	4 (50%)
White	21 (22%)	21 (23%)	9 (17%)	2 (25%)
Multiracial	2 (2%)	2 (2%)	0 (0%)	0 (0%)
Native Hawaiian/Pacific Islander	1 (1%)	1 (1%)	1 (2%)	2 (25%)
Missing	1 (1%)	1 (1%)	1 (2%)	0 (0%)
Ethnicity				
Not Hispanic	51 (54%)	50 (54%)	27 (51%)	5 (63%)
Hispanic	43 (46%)	43 (46%)	26 (49%)	3 (37%)
Treatment Group				
Intervention	50 (53%)	49 (53%)	26 (49%)	5 (63%)
Waitlist Control	44 (47%)	44 (47%)	27 (51%)	3 (37%)

**Fig. 2:**
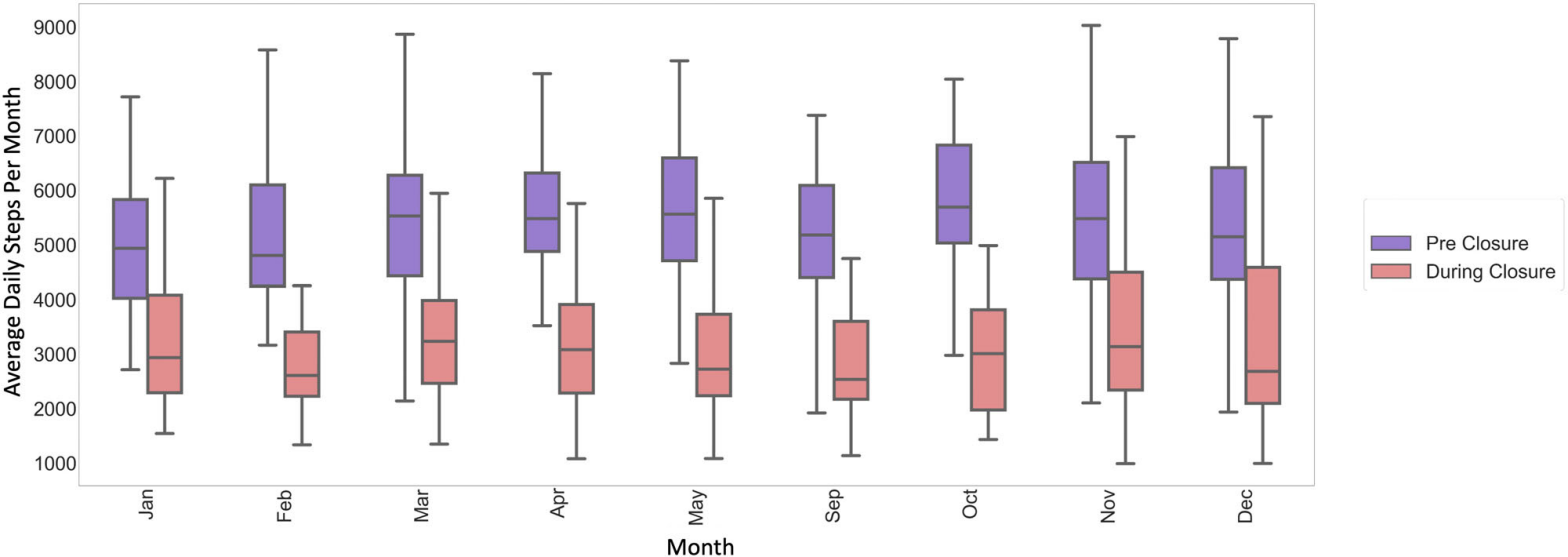
Average daily step count per month between 7 am and 4 pm. A decrease of 1970 average daily steps was observed during the times children were expected to be in school. The purple and pink boxes indicate the pre- and during-closure months respectively. The center line represents the median of the data and the lower and upper edges of the box correspond to the first and third quartiles, respectively. The whiskers extend from the box by 1.5 times the inter-quartile range.

Pre-closure school-time daily step count ranged from 5085 to 5789 steps (average 5441 ± 229) with an overall average daily step count of 8889 for in-school months. This step count indicates that roughly 61% of daily steps can be attributed to activity likely occurring during school hours. During-closure, we found that daily step count during school hours declined by 1973 steps (average 3468 ± 542, *n* = 52) (*p* < 0.05) (Fig. 2). The post-closure school-time step count averaged 4616 ± 39 steps (*n* = 8).

**Fig. 3:**
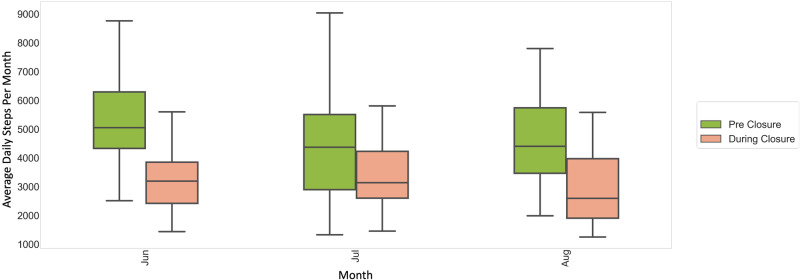
Average daily step count per month during weekdays between 7 am and 4 pm during the summer months. A decrease of 1537 average daily steps was observed during school-times in the summer of 2020 as compared to 2019. The green and orange boxes indicate the pre- and during-closure months respectively. The center line represents the median of the data and the lower and upper edges of the box correspond to the first and third quartiles, respectively. The whiskers extend from the box by 1.5 times the inter-quartile range.

Additionally, we observed an average decrease of 1537 steps during what would be school times during weekdays in the summer months of 2020 as compared to the summer of 2019. Average pre-closure school-time summer daily step count was 4888 steps (*n* = 44), while the average during-closure school-time summer daily step count declined to 3351 steps (*n* = 41) (*p* < 0.05). The average daily step counts during school times were also lower during the summer as compared to the rest of the year for both pre-closure (5441 vs 4888 steps) and during-closure (3468 vs 3351 steps) but the difference in step counts for summer compared to the rest of the year was more pronounced pre-closure. The post-closure school-time step count during the summer was 4487 steps (*n* = 4) (Fig. 3).

### Sleep

**Fig. 4:**
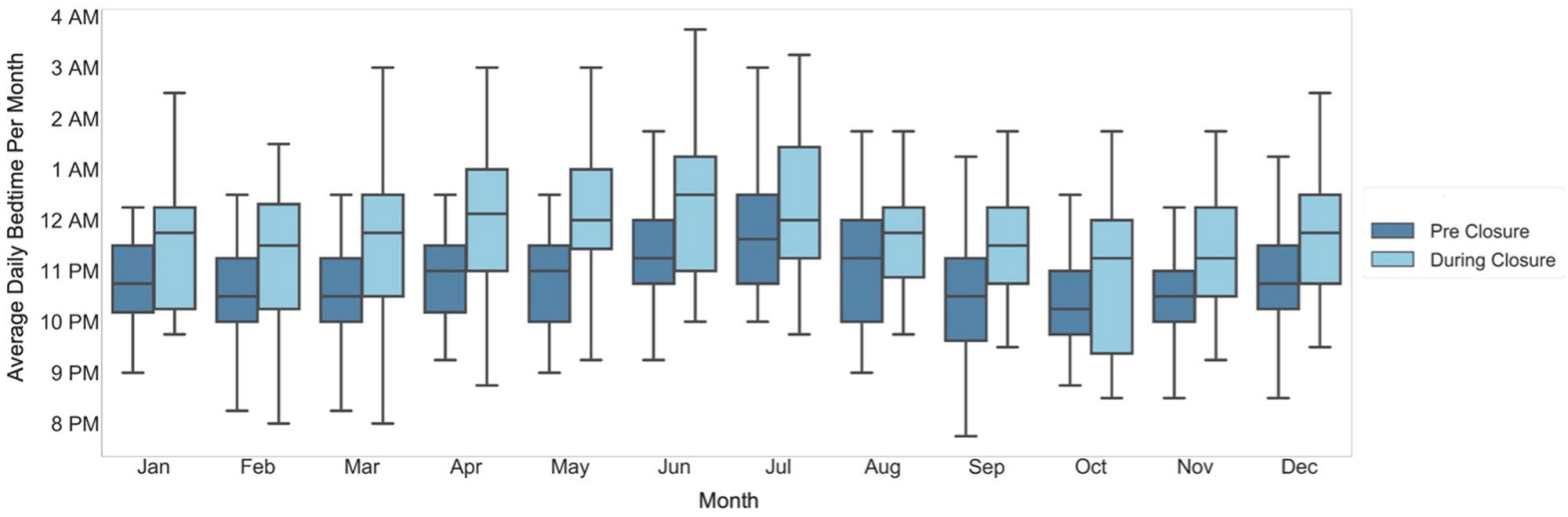
Average daily bedtime pre-closure and during-closure. The dark blue and light blue boxes indicate the bedtime for the pre-closure and during-closure months, which are shown side by side for comparison. The center line represents the median of the data and the lower and upper edges of the box correspond to the first and third quartiles, respectively. The whiskers extend from the box by 1.5 times the inter-quartile range.

**Fig. 5:**
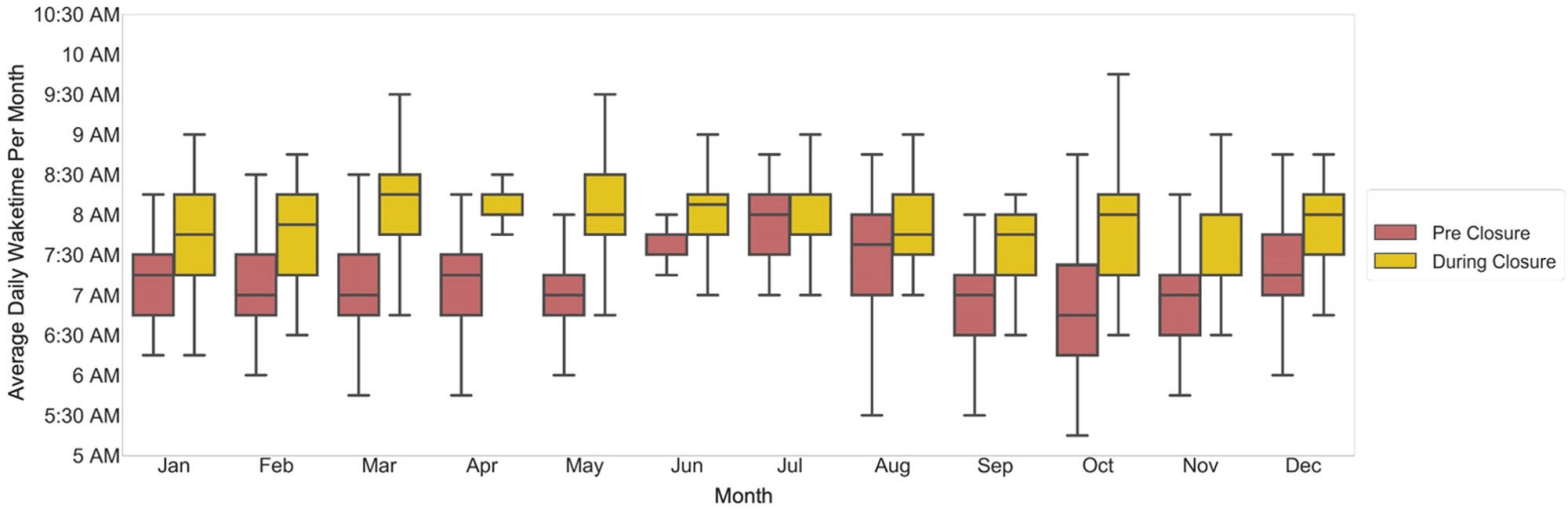
Average daily wake time pre-closure and during-closure. The red and gold boxes show the monthly values for pre-closure and during-closure months, respectively. The center line represents the median of the data and the lower and upper edges of the box correspond to the first and third quartiles, respectively. The whiskers extend from the box by 1.5 times the inter-quartile range.

**Fig. 6:**
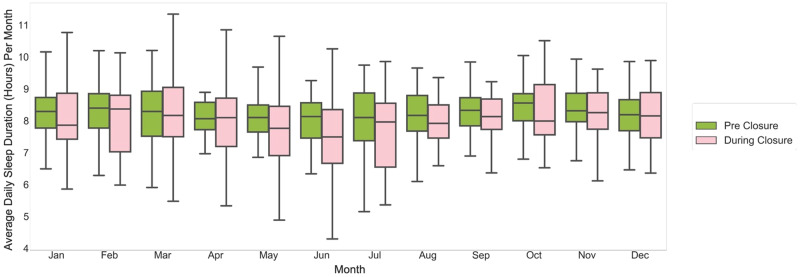
Average daily sleep duration pre-closure and during-closure. The green and pink boxes show the sleep durations for pre-closure and during-closure months, respectively. The center line represents the median of the data and the lower and upper edges of the box correspond to the first and third quartiles, respectively. The whiskers extend from the box by 1.5 times the inter-quartile range.

We observed a delay in sleep-onset time during-closure (average 11:45 pm ± 32 min overall, 12:15 am ± 17 min in summer months) as compared with pre-closure (average 10:45 pm ± 25 min overall, 11:30 pm ± 17 min in summer months) (Fig. 4). Similarly, we observed a delay in wake time during-closure (average 8:00 AM ± 11 min overall; 8:00 AM ± 3 min in summer months) as compared with wake time pre-closure (average 7:15 AM ± 16 min overall; 7:30 AM ± 10 min in summer months) (Fig. 5). Overall sleep duration during-closure decreased by 12 min (0.2 h) as compared with pre-closure, from 8.1 ± 0.17 h on average to 7.9 ± 0.22 (*p* = 0.01) (Fig. 6).

## Discussion

This study examined the PA and sleep behaviors of a cohort of children and adolescents with obesity during the COVID-19 pandemic. Children enrolled in this program aimed at reducing childhood obesity, experienced a sudden and significant decrease in objectively-measured PA and sleep. These changes correlated in time with COVID-19 related school closures, and started trending back toward pre-closure levels when schools reopened. Specifically, we found a group-level decrease in average daily step count of over 1655 steps (19% decrease), delays in sleep onset and waketime by about 1 h and 45 min, respectively, and decrease in overall sleep duration by 12 min (0.2 h) on an average (a decrease of 2.5%), during-closure compared to pre-closure.

Several studies assessing changes in PA and sleep habits in children and adolescents as public health guidelines were implemented and structured school activities were suspended during the COVID-19 pandemic found similar disruption of PA habits and sleep routines globally. Median time spent in PA decreased by 435 min/week in children (*n* = 2426) in Shanghai, China, based on self-reported PA^29^. Another study reported reduced PA by 91 min/day in March–April 2020 as compared to September-December 2019, as assessed using online questionnaires in a cohort of Spanish children^30^. Additionally, multiple scoping and systematic reviews of studies across the globe reported reductions in PA time, later bed and wake times, and increased sleep duration among children and adolescents between the ages of 1–19 years, where majority of the included studies used self-reported data and fewer studies used accelerometer data^24–27^. Reported PA changes ranged anywhere from 10 min decrease per day, up to 90 min decrease per day^30–32,34,36^, with studies reporting that factors such as pre‐pandemic PA practices, socio‐economic status, race and, age influenced PA during the pandemic^26,48–50^.

Similar findings emerged among studies specifically using objectively measured PA modalities, such as Actigraph, in addition to commercial wearables^31–34,36,37^. In the US, a study of 168 Texas school children reported a 9.4 min decrease in mean daily MVPA using 7 days of Actigraph data^31^. Similarly, in Italian primary school children, daily MVPA minutes declined by over 15 min, and weekly MVPA minutes were reduced by over 30 min, based on Actigraph data from one week each of pre and post pandemic onset^32^. MVPA was also assessed among children in the UK, separated by weekday and weekend day activity^33^. Salway et al. found that weekday and weekend MVPA declined by 7.7 and 6.9 min, respectively, in 2021 compared to 2018 data, using 5 days of accelerometer data from comparable groups^33^. Similarly, a study reported a PA decrease of 43.3 min per day using accelerometer data collected three days prior to lockdown and during the lockdown each in 21 Spanish preschoolers^34^. A quasi‐experimental interrupted time series study reported decreased MVPA by about 10 min per day over 6 weeks in spring in 2020 in comparison to changes in springs of 2018 and 2019, as measured by Fitbit Charge‐2 in 231 children^36^. Another study found a 21%-24% decrease in step counts in late March and early April 2020 among children with congenital heart disease in Canada using Fitbit Charge 2 device^37^.

While the majority of literature assesses PA in minutes, there is a lack of research assessing changes in actual step count during the pandemic, which reduces the ability to reasonably compare the data we collected with that from other studies. However, based on a review comparing step count to minutes of activity among children, a decrease in steps in the current study by over 1600 steps seems to be consistent with the findings of 7–15 min per day reductions in activity reported by other studies using accelerometer and other objective measures^51^.

Several studies also documented changes in sleep habits in children during the COVID-19 pandemic^24,28,34,36^. Sleep time increased by 0.65 ± 1.29 h/day, three weeks into the pandemic-associated closures, in 41 children with obesity in Italy based on self-reported data^28^. Burkart et al. reported delayed sleep midpoints by over 124 min and increased sleep duration of 17 min using Fitbits in 231 children over 6 weeks of spring in 2020 in comparison to change in springs of 2018 and 2019 in an interrupted time series study design^36^. Alonso-Martínez et al. reported reduced sleep efficiency (defined as the “percent of minutes scored as sleep between onset and offset”) by 2.09% in 21 Spanish preschoolers using accelerometer data collected three days prior to lockdown and three days during the lockdown^34^.

Our findings of reduced activity overall and especially during school times during- pandemic likely point to a relationship between remote learning and sedentary time, and intuitively suggest a reduction in PA that would occur during in-person school such as walking to and from school, physical education or recess sessions, and walking between classrooms. While these data represent an association and cannot prove causation, school-based activities have been shown to help children achieve daily MVPA goals and maintain timely sleep routines^52^. The strategies associated with preventing the spread of COVID-19 exposed children to longer-than-normal periods of reduced structure, which demonstrated how structured programs, such as those provided by in-person schooling, impact health behaviors among children^53^. Structured weekday activities during school days induce healthy PA, sleep regularity, and eating behaviors in children as compared to vacation days or weekends and this might contribute to increased weight gain in children over the summer months as compared with the school year, as documented by some studies^3,7^. The timing of PA and sleep habit changes reported here correlates very closely with the local school closures and re-openings. Given these factors, we believe that these important health behavior changes may have been an unintended consequence of the disruption of daily routines caused by measures that closed schools and recreational activities during the COVID-19 pandemic.

The behavioral changes we observed are critically important to child health and may have been especially damaging to children with pre-pandemic obesity and cardiometabolic co-morbidities. For example, PA is both a prevention and a treatment strategy for excess adiposity in children. The World Health Organization (WHO) conducted a systematic review in 2020, and found strong evidence that at least 60 min of MVPA per day is associated with lesser adiposity, improved cardiometabolic health, and important cognitive-related outcomes including mood, academic performance, and quality of life^54^. The American Academy of Pediatrics recommends that children with obesity increase their energy expenditure, which may reduce or maintain BMI, and also may improve cardiometabolic health even in the absence of weight loss^55^.

Children’s sleep habits are also known to be related to overall health^56,57^. An international systematic review including over 500,000 participants from 40 countries showed that longer sleep duration was associated with lower adiposity, better emotional regulation, better academic achievement, and better quality of life^56^. Further, there is a positive association between short sleep duration and greater BMI in children^2^; and healthy sleep, activity, and eating routines are all key components of obesity treatment in children^58^. Hence, the changes observed in this study in both PA and sleep habits during-pandemic are especially worrisome and emphasize the importance of structured routines for maintaining healthy PA and sleep levels in children with obesity.

This study has strengths and limitations. This study represents a secondary data analysis of the H&P clinical trial, which was collecting objectively-measured PA and sleep data when the COVID-19 pandemic occurred. Data collection was remote, using the wearable Garmin devices; thus measures were able to be continually tracked over the period of the COVID-19 pandemic. Uniquely, this allowed investigators to have the ability to correlate changes in daily PA and sleep with local policies that established mitigation measures, including school closures.

Compared to subjective data, data collected from fitness trackers over time is a more reliable indicator of changes in PA and sleep during the pandemic. A major advantage of this study is the availability of continuous, longitudinal data collected from 94 children starting from 2019 and continuing into 2021, demonstrating longitudinal changes in PA and sleep in children and adolescents with obesity during the COVID-19 associated lockdown measures in the US, as objectively measured from commercial fitness trackers.

The study population was comprised of children and adolescents with obesity enrolled in an intervention study for encouraging healthier lifestyle and PA in Durham, NC. A limitation of this analysis is the availability of data from 94 children and adolescents with obesity from the Durham, NC region only, some of whom were part of the intervention arm of the H&P program, which could have impacted their PA. The number of participants whose data were available for post-closure analysis was also much smaller compared to the overall cohort size. This was because the participants were enrolled for 1 year in the study and the enrollment ended right before the start of the pandemic. Finally, while fitness trackers provide objective measures of activity as compared with more subjective self-reports, there have been concerns around the accuracy of commercial fitness trackers^59^. There are also very few validation studies for the Garmin Vívofit 3 in children. One particular challenge for this study is that the tracker did not differentiate between non-wear and sedentary times. However, contextual data was employed to address this concern as described in the methods.

This study suggests there was a decline in PA and disturbed sleep habits among children with obesity during the COVID-19 pandemic. This trend continued for almost a year, with seeming improvements in activity habits back to pre-closure normal with the reopening of schools, pointing to a possible association between structured school activities and healthy PA levels. Findings support the importance of structured, daily routines on promoting health behaviors and, as such, may inform future policy decisions about school and extracurricular activity closures. With this study, we provide information to the community, including teachers, clinicians, and policy makers on how interruptions to normal daily routines including in-person school and activities may impact children. Our results should provide motivation for increasing opportunities for children, especially for those with obesity, for structured activities to promote adherence to routines and prevent unhealthy PA and sleep habits during school closures, such as summer months.

## Methods

This study is a secondary data analysis of the Hearts & Parks (H&P) crossover randomized controlled trial (ClinicalTrials.gov NCT03339440), a clinic-community collaboration targeted towards reducing childhood obesity among children in North Carolina (NC). Participants were enrolled between February 8, 2018 and March 10, 2020^60^. However, to ensure similar time duration for the statistical comparison between pre-closure and during-closure values, we only include data starting March 1, 2019 for this analysis. Prior to the pandemic, H&P enrolled and randomized 260 children and adolescents with obesity into either the 6-month clinic-community intervention or a waitlist control group, who received usual care until they entered the intervention at 6 months. In the intervention, patients received care at a pediatric weight management program and were able to participate in a structured play and exercise program, Bull City Fit, delivered at a local parks and recreation center. In Bull City Fit (offered 6 days/week), participants engaged in 60 min of PA at every session and were offered weekly nutrition education. The waitlist control group was a 6-month waitlisted group, where participants received a non-obesity-related literacy intervention during the first 6 months, after which they were invited to participate in the intervention for the remaining 6 months. The study was approved by the Duke Health Institutional Review Board (IRB# Pro00086684) and was funded by the American Heart Association (AHA) Strategically Focused Research Network 17SFRN33670990. All participants provided assent, and their parent or legal guardian provided written consent for study participation.

The COVID-19 pandemic led to the closure of Bull City Fit in-person sessions as well as in-person school closures beginning on March 15, 2020. For the purposes of our analysis, we define “pre-closure” to be the period between March 1, 2019 and March 14, 2020 (~12 months), “during-closure” to be the period between March 15, 2020 and March 31, 2021 (~12 months), and “post-closure” to be the period between April 1, 2021 and June 30, 2021 (~3 months). These time frames were chosen based on the announcement of stay-at-home orders for North Carolina (announced on Saturday, March 14, 2020)^61^, and when most schools in the Durham school district returned to in person learning (April 2021). The inclusion criteria for H&P required that they live in a geographic radius such that the majority of, if not all, children would be attending a public, private, or charter school in Durham County.

### Outcomes

The primary outcomes of interest were PA (defined as step count), bedtime, waketime, and sleep duration pre-, during-, and post- the COVID-19-associated closures.

### Physical activity

PA was measured objectively using step counts from a water-resistant Garmin VivoFit 3 wristband, chosen for its long battery life to last for the study duration without the need for charging (1-year battery life)^62^. The Garmin VivoFit 3 has been reported to accurately monitor step counts in children through multiple research studies, which demonstrated, for example, equivalence to pedometer-measured step counts^63,64^. The Garmin VivoFit 3 has also been shown to perform at <5% mean absolute percentage error for laboratory-based treadmill study over the range of normal speeds in adults^65^. Participants were instructed to wear the watch 24 h a day for the entire one-year study duration. The Garmin Connect app was downloaded and set up on participants’ or their parents’ smartphones. Parents and/or participants were instructed to sync the smartwatches to the app at least once a week. Garmin data were collected and aggregated by Pattern Health Technologies, Inc., who provide digital health platforms to manage health programs. Fifteen-minute epoch-level (where epoch is the time interval for which step count information was provided) step count information was transformed into daily step counts, which was then used to calculate average daily step counts per month (available for *n* = 252). Zero values were not reported by Pattern Health or Garmin, and thus it was not possible to differentiate non-wear time from sedentary times from the step count data alone. To address this, we leveraged mean motion intensity (MMI), a metric reported by the Garmin device, as a proxy for watch wear. MMI is a proprietary measure provided for each activity epoch that can take on values between 0 and 7, where 0 corresponds to no activity intensity and 7 corresponds to the greatest measurable activity intensity. We defined non-wear as MMI < 1 and used this definition to remove data corresponding to non-wear times from analysis, which resulted in data availability for 218 participants.

From there, the minimum daily valid wear time and the threshold for number of days with step count data needed for inclusion in the analysis were determined through a combination of literature and data-driven decisions that were fit for the purpose of this study and data analysis. Participants were included in the analysis if they had sufficient step count data covering the entire pre-, during-, and post-closure period between March 1, 2019 and June 30, 2021. For an accurate overview of monthly step counts, a 60-day threshold was chosen to ensure adequate representation of each participant’s step count trends across different closure periods and to prevent bias in data patterns from participants with fewer data points (Supplementary Fig. 1). This decision was informed by the histogram shown in Supplementary Fig. 1, which allowed for a visual determination of a valid wear days cutoff that would provide enough data from participants who wore their smart watches regularly while improving confidence in the results and bolstering the reliability of our findings. A similar approach was used by Rezaei et. al to set a cutoff of 10-days to establish habitual sleep patterns for a month from wearable data for understanding sleep-related changes in adults at the population level, partially based on visual inspection of frequency of use to determine regular usage of wearable devices^66^.

While typical accelerometer-based studies have a duration of 4–7 days of monitoring^67,68^, longitudinal studies that employ commercial fitness trackers aim to capture trends over months or even years. As such, the data quality control methods vary between short-duration and longitudinal studies. It is worth noting that multi-year longitudinal studies employing commercial smartwatches remain relatively new, and thus there is insufficient literature in existence for best practices surrounding data processing decisions.

For the purpose of our study, a day was considered valid if the participant had more than 41% wear time (the presence of more than 40 out of the 96 possible epochs that can be reported in 24 h, which is equivalent to more than 600 min, or 10 h, per day), These definitions for daily wear-time criteria have been used in previous studies with accelerometers^69,70^. Hence, we only included data for participants who had more than 60 days of valid data, and only considered valid days for analysis.

The application of inclusion criteria resulted in data from 94 participants included in the final PA analysis presented here (female: 55.3%, median age: 9.7 years) (Supplementary Figs. 1 and 2; Table 1), with *n* = 93 (female: 54.8%, median age: 9.7 years), *n* = 53 (female: 52.8%, median age: 9.8 years) and *n* = 8 (female: 50%, median age: 8.8 years) for pre-closure, during-closure, and post-closure, respectively. The post-closure sample size was fairly small in our analysis but we report average PA and sleep metrics post-closure to offer a potential trend in PA and sleep patterns directions.

To account for activities that occurred during school hours, we also explored step counts only during the times when children were expected to be in school (7:00 AM–4:00 PM on non-summer weekdays). Step count values were extracted on weekdays for all months excluding the summer months of June, July and August, when schools are typically on break.

### Sleep

Sleep was measured objectively using the Garmin wristband. Sleep epochs were detected by Garmin’s proprietary sleep detection algorithm, and reported in seconds. Sleep epochs varied in duration as expected based on the number of sleep episodes detected and their individual durations^71^. Some nights had multiple sleep epochs for single participants, indicating disturbed sleep, movement during sleep, improper watch wear during sleep, or algorithmic error. The sleep periods labeled by Garmin were used for the sleep duration, bedtime, and waketime analysis.

### Data/statistical analysis

Step data were aggregated at the daily level for each participant by summing the step count within each epoch for each day only for epochs with an associated MMI ≥1. The average daily steps per month were calculated for each participant, and then averaged across all participants to calculate the average daily step count per month for the entire cohort. These average values and their standard deviations were reported for each of the three time periods: pre-closure, during-closure and post-closure (*n* = 93, 53, and 8, respectively), to enable comparison of PA during each period and to explore whether a relationship would emerge between PA and closures. For analysis of PA during school times, step count observations beginning from 7:00 AM and before 4:00 PM on weekdays, for all months except June, July and August, were included. Daily step count (averaged by month) for school times were calculated in the same way as the overall step count analysis described above (*n* = 93, 52 and 8, respectively for pre-closure, during-closure and post-closure).

For the sleep analysis, we used the time stamp of the earliest sleep epoch recorded after 6:00 PM on that day and 8:00 AM the following day to define the sleep start time, or bedtime. Similarly, we defined waketime as the latest sleep end time among all such epochs. If the bedtime was after midnight, the bedtime date was adjusted to be the date for the previous day. Total daily sleep duration per participant was calculated as the sum of the durations of all sleep epochs for each day. Average bedtime, waketime, and sleep duration per participant were calculated for each month, and these values were averaged for all participants to obtain the overall population average. Average bedtime, and waketime were each rounded to the nearest 15 min and were reported, along with the standard deviations, for each of the three time periods: pre-closure, during-closure and post-closure. Additionally, we report summer bedtime and waketime values separately to emphasize similarities in trends during typical summer pre-closure and both school-year and summer months during-closure.

We performed the Mann–Whitney *U* Test to determine whether step count and sleep duration were significantly changed between the pre- and during-closure periods. To account for variations that are potentially attributable to seasonal changes, pre-closure step counts and sleep durations were compared with the corresponding months during-closure. Given that the post-closure data were only available for a small number of participants (N = 8) and for a limited number of months, we exclude the post-closure data from the statistical analysis and only report the average values for all PA and sleep metrics post-closure.

All analyses were conducted using Python version 3.7.4 through Jupyter notebooks (Jupyter notebook 6.0.1) in the Duke Protected Analytics Computing Environment (PACE) given the sensitive nature of the data. The visualizations were generated using the Seaborn library in Python version 3.7.4.

### Reporting summary

Further information on research design is available in the Nature Research Reporting Summary linked to this article.

### Supplementary information

Supplementary Information

Reporting Summary

## Data Availability

Aggregate step count and sleep data analyzed in this study may be made available upon reasonable request by contacting the corresponding author via the e-mail address provided.

## Code availability

The code used for this manuscript is available on Github (https://github.com/KarnikaSingh/PA-Sleep-Analysis).
