# Supplementary material for: Physical activity and sleep changes among children during the COVID-19 pandemic
Source: NPJ Digit Med. 2024 Mar 16;7:70. doi: 10.1038/s41746-024-01041-8 (PMC10944532; doi:10.1038/s41746-024-01041-8)
Supplement: Supplementary file 1 — Supplementary Information [file 41746_2024_1041_MOESM1_ESM.pdf]

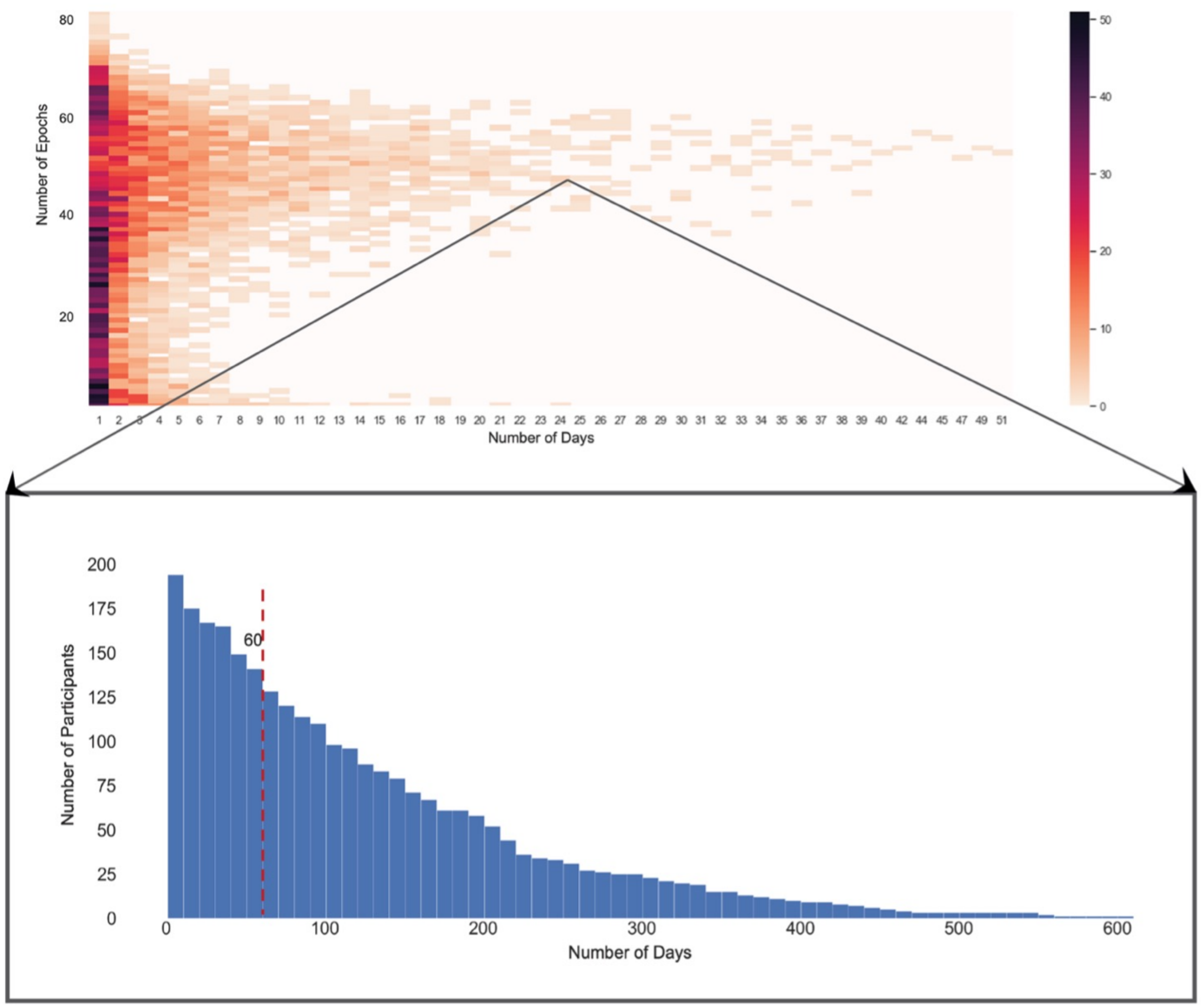

Supplementary Figure 1: Data availability. The heatmap at the top shows the spread of epoch numbers on the y-axis for which data was available for the corresponding number of days on the x-axis for all individuals. The histogram at the bottom shows the number of days of data availability for the participants. The y-axis shows the number of participants for whom data with >40 epochs is available for the corresponding number of days on the x-axis.

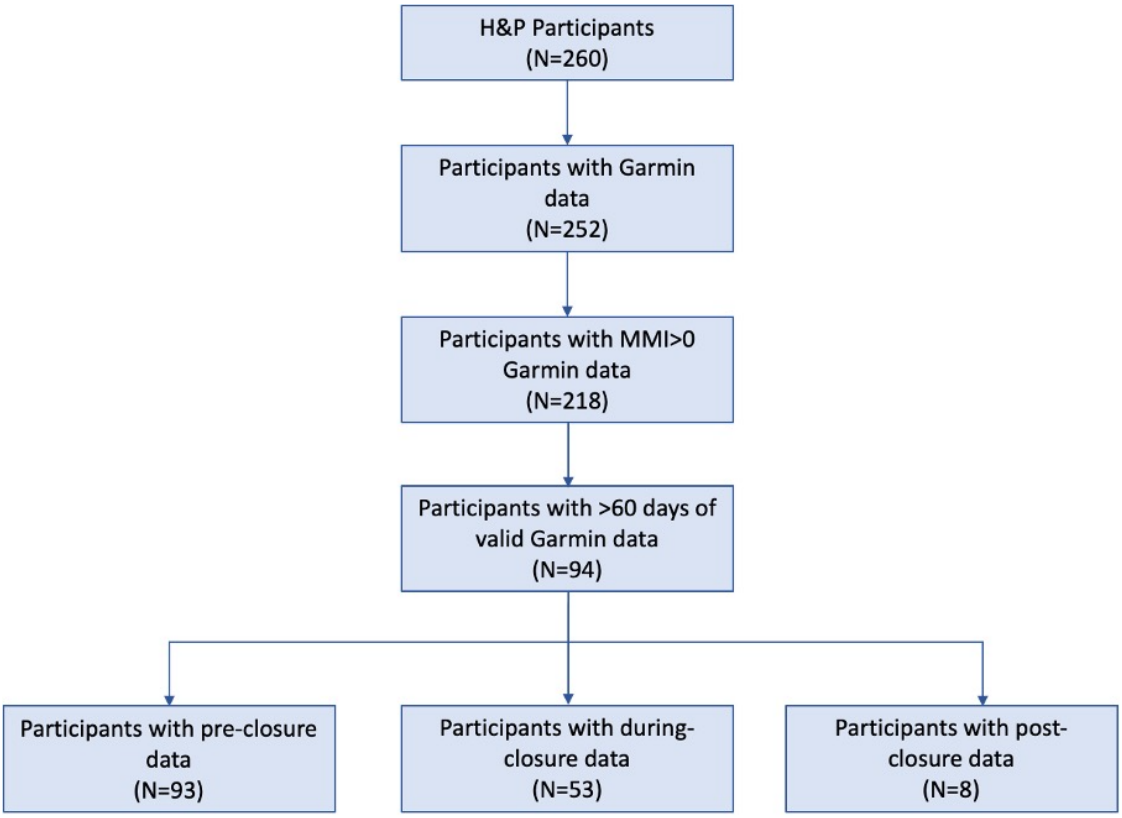

Supplementary Figure 2: Flow chart of the inclusion and exclusion criteria
