# Supplementary material for: Physical activity and sleep changes among children during the COVID-19 pandemic
Source: NPJ Digit Med. 2024 Mar 16;7:70. doi: 10.1038/s41746-024-01041-8 (PMC10944532; doi:10.1038/s41746-024-01041-8)
Supplement: Supplementary file 2 — Reporting Summary [file 41746_2024_1041_MOESM2_ESM.pdf]

nature portfolio

Corresponding author(s): *Double-anonymous peer review submissions: write DAPR and your manuscript number here instead of author names.*

Last updated by author(s): *YYYY-MM-DD*

Reporting Summary

Nature Portfolio wishes to improve the reproducibility of the work that we publish. This form provides structure for consistency and transparency in reporting. For further information on Nature Portfolio policies, see our Editorial Policies and the Editorial Policy Checklist.

Statistics

For all statistical analyses, confirm that the following items are present in the figure legend, table legend, main text, or Methods section.

n/a	Confirmed	
☐	☒	The exact sample size (*n*) for each experimental group/condition, given as a discrete number and unit of measurement
☐	☒	A statement on whether measurements were taken from distinct samples or whether the same sample was measured repeatedly
☐	☒	The statistical test(s) used AND whether they are one- or two-sided<br>*Only common tests should be described solely by name; describe more complex techniques in the Methods section.*
☐	☒	A description of all covariates tested
☒	☐	A description of any assumptions or corrections, such as tests of normality and adjustment for multiple comparisons
☐	☒	A full description of the statistical parameters including central tendency (e.g. means) or other basic estimates (e.g. regression coefficient) AND variation (e.g. standard deviation) or associated estimates of uncertainty (e.g. confidence intervals)
☐	☒	For null hypothesis testing, the test statistic (e.g. *F*, *t*, *r*) with confidence intervals, effect sizes, degrees of freedom and *P* value noted<br>*Give P values as exact values whenever suitable.*
☒	☐	For Bayesian analysis, information on the choice of priors and Markov chain Monte Carlo settings
☒	☐	For hierarchical and complex designs, identification of the appropriate level for tests and full reporting of outcomes
☒	☐	Estimates of effect sizes (e.g. Cohen's *d*, Pearson's *r*), indicating how they were calculated

Our web collection on statistics for biologists contains articles on many of the points above.

Software and code

Policy information about availability of computer code

Data collection	This study is a secondary data analysis of the Hearts & Parks (H&P) crossover randomized controlled trial (ClinicalTrials.gov NCT03339440), a clinic-community collaboration targeted towards reducing childhood obesity among children in North Carolina (NC). Participants were enrolled between February 8, 2018 and March 10, 2020. H&P enrolled 260 children and adolescents with obesity. The study was approved by the Duke Health Institutional Review Board (IRB# Pro00086684) and was funded by the American Heart Association (AHA) Strategically Focused Research Network 17SFRN33670990. Physical Activity and sleep was measured objectively using step counts from a Garmin VivoFit 3 wristband. All participants provided assent, and their parent or legal guardian provided consent, for study participation.
Data analysis	All analyses were conducted using Python version 3.7.4 through Jupyter notebooks (Jupyter notebook 6.0.1) in the Duke Protected Analytics Computing Environment (PACE) given the sensitive nature of the data. The visualizations were generated using the Seaborn library in Python version 3.7.4.<br>The code used for this manuscript is available on Github(https://github.com/KarnikaSingh/PA-Sleep-Analysis).

For manuscripts utilizing custom algorithms or software that are central to the research but not yet described in published literature, software must be made available to editors and reviewers. We strongly encourage code deposition in a community repository (e.g. GitHub). See the Nature Portfolio guidelines for submitting code & software for further information.

Data

Policy information about availability of data

All manuscripts must include a data availability statement. This statement should provide the following information, where applicable:

- Accession codes, unique identifiers, or web links for publicly available datasets
- A description of any restrictions on data availability
- For clinical datasets or third party data, please ensure that the statement adheres to our policy

Aggregate step count and sleep data analyzed in this study may be made available upon reasonable request by contacting the corresponding author via the e-mail address provided.

Research involving human participants, their data, or biological material

Policy information about studies with human participants or human data. See also policy information about sex, gender (identity/presentation), and sexual orientation and race, ethnicity and racism.

Reporting on sex and gender	Nearly 55% participants included in the analysis were female. Gender was reported by the participants. We do not perform any gender-based analysis and only report results for the overall population.
Reporting on race, ethnicity, or other socially relevant groupings	Nearly 54% participants included in the analysis were not Hispanic and 34% were African American. We report the race and ethnic distribution of our population group. These are based on self-reports by the participants during study recruitment. We do not perform any race or ethnicity based analysis. We only report results for the overall population.
Population characteristics	Of the 94 participants included in this analysis, 52 (55%) were female, 65 (69%) were in the age group 5-10 years (median age 9.7 ±3.1), and 54% participants were not Hispanic. We do not perform any specific analysis based on age, race or gender and only present overall population-level results.
Recruitment	This study is a secondary data analysis of the Hearts & Parks (H&P) crossover randomized controlled trial (ClinicalTrials.gov NCT03339440), a clinic-community collaboration targeted towards reducing childhood obesity among children in North Carolina (NC). Participants were enrolled between February 8, 2018 and March 10, 2020.
Ethics oversight	The study was approved by the Duke Health Institutional Review Board (IRB# Pro00086684) and was funded by the American Heart Association (AHA) Strategically Focused Research Network 17SFRN33670990. All participants provided assent, and their parent or legal guardian provided consent, for study participation.

Note that full information on the approval of the study protocol must also be provided in the manuscript.

Field-specific reporting

Please select the one below that is the best fit for your research. If you are not sure, read the appropriate sections before making your selection.

☐ Life sciences ☒ Behavioural & social sciences ☐ Ecological, evolutionary & environmental sciences

For a reference copy of the document with all sections, see nature.com/documents/nr-reporting-summary-flat.pdf

Behavioural & social sciences study design

All studies must disclose on these points even when the disclosure is negative.

Study description	Observational longitudinal cohort study.
Research sample	260 children and adolescents with obesity were enrolled in the study for one year. Participants wore a Garmin activity tracker for the duration of the study. The Garmin data was used for physical activity and sleep analysis.
Sampling strategy	Hearts & Parks enrolled and randomized 260 children and adolescents with obesity into either the 6-month clinic-community intervention or a waitlist control group, who received usual care until they entered the intervention at 6 months. In the intervention, patients received care at a pediatric weight management program and were able to participate in a structured play and exercise program, Bull City Fit, delivered at a local parks and recreation center. In Bull City Fit (offered 6 days/week), participants engaged in 60 minutes of PA at every session and were offered weekly nutrition education. The waitlist control group was a six-month waitlisted group, where participants received a non-obesity-related literacy intervention during the first six months, after which they were invited to participate in the intervention for the remaining six months.
Data collection	All participants wore a Garmin activity tracker for the duration of the study. The Garmin data was used for physical activity and sleep analysis.
Timing	For this analysis, data collected between March 1, 2019 and June 30, 2021 was used. For the purposes of our analysis, we define "pre-closure" to be the period between March 1, 2019 and March 14, 2020 (~12 months), "during-closure" to be the period between

	March 15, 2020 and March 31, 2021 (~12 months), and "post-closure" to be the period between April 1, 2021 and June 30, 2021 (~3 months).
Data exclusions	Garmin data was available for 252 participants. We removed data corresponding to non-wear times from analysis, which resulted in data availability for 218 participants. We only included data for participants who had more than 60 days of valid data, and only considered valid days for analysis (where valid day was defined as one with more than 10 hours of data). This resulted in data from 94 participants included in the final PA analysis.
Non-participation	We are using the Garmin data available from all Hearts & Parks participants.
Randomization	Observational longitudinal study design was employed to investigate the research question. Therefore, no randomization was performed.

Reporting for specific materials, systems and methods

We require information from authors about some types of materials, experimental systems and methods used in many studies. Here, indicate whether each material, system or method listed is relevant to your study. If you are not sure if a list item applies to your research, read the appropriate section before selecting a response.

Materials & experimental systems

n/a	Involved in the study
☒	☐ Antibodies
☒	☐ Eukaryotic cell lines
☒	☐ Palaeontology and archaeology
☒	☐ Animals and other organisms
☒	☐ Clinical data
☒	☐ Dual use research of concern
☒	☐ Plants

Methods

n/a	Involved in the study
☒	☐ ChIP-seq
☒	☐ Flow cytometry
☒	☐ MRI-based neuroimaging

Plants

Seed stocks	N/A
Novel plant genotypes	N/A
Authentication	N/A
